# Cucumber mosaic virus-induced gene and microRNA silencing in water dropwort (*Oenanthe javanica* (Blume) DC)

**DOI:** 10.1186/s13007-023-01129-4

**Published:** 2024-01-11

**Authors:** Zhen He, Shuangyu Sheng, Lingqi Wang, Tingting Dong, Kun Zhang, Liangjun Li

**Affiliations:** 1 College of Plant Protection, Yangzhou University, Wenhui East Road No.48, Yangzhou, Jiangsu Province 225009 People’s Republic of China; 2 College of Horticulture and Landscape Architecture, Yangzhou University, Yangzhou, People’s Republic of China

**Keywords:** *Oenanthe javanica*, Cucumber mosaic virus (CMV), Virus-induced gene silencing (VIGS), *Gibberellin-insensitive dwarf 1*, miRNA

## Abstract

Water dropwort (*Oenanthe javanica* (Blume) DC), an aquatic perennial plant from the Apiaceae family, rich in dietary fibert, vitamins, and minerals. It usually grows in wet soils and water. Despite accumulating the transcriptomic data, gene function research on water dropwort is still far behind than that of the other crops. The cucumber mosaic virus (CMV) induced gene silencing was established to study the functions of gene and microRNA (miRNA) in the water dropwort. CMV Fast New York strain (CMV-Fny) genomic RNAs 1, 2, and 3 were individually cloned into pCB301 vectors. We deleted part of the ORF 2b region and introduced recognition sites. A CMV-induced gene silencing vector was employed to suppress the expression of endogenous genes, including *phytoene desaturase* (*PDS*). In order to assess the efficacy of gene silencing, we also cloned conserved sequence of *gibberellin insensitive dwarf (GID1)* cDNA sequences into the vector and inoculated the water dropwort. The height of CMV-*GID1*-infected plants was marginally reduced as a result of *GID1* gene silencing, and their leaves were noticeably longer and thinner. Additionally, we also used a CMV-induced silencing vector to analyze the roles of endogenous miRNAs. We used a short tandem target mimic approach to clone miR319 and miR396 from water dropwort into the CMV vector. Plants with CMV-miRNA infection were driven to exhibit the distinctive phenotypes. We anticipate that functional genomic research on water dropwort will be facilitated by the CMV-induced gene silencing technique.

**Supplementary Information:**

The online version contains supplementary material available at 10.1186/s13007-023-01129-4.

## Introduction

Water dropwort (*Oenanthe javanica* (Blume) DC) is a perennial aquatic herb belonging to the genus *Oenanthe* of the Apiaceae family [1]. It is commonly grown in East Asian nations such South Korea, China, and Japan [2]. Having a large customer base due to its high vitamin, protein, dietary fiber, and mineral nutrient content is water dropwort, a well-liked vegetable [3, 4]. It also has various medicinal benefits such as, antithrombotic [5], hepatoprotective [6], neuroprotective [7], anti-inflammatory [8], antioxidant [9], antiviral [10], and antisenescence effects [11]. Moreover, recent years have witnessed increasing research on water dropwort. Despite the fact that Feng created a complete water dropwort transcriptome using PacBio SMRT and Illumina RNA sequencing, research on its gene function is fairly limited compared to other crops [12]. However, the genetic transformation, gene silencing, and gene knockout systems of water dropwort have not been established, along with a lack of corresponding technical support for gene function research. It is necessary that the genetic tools will be developed to address these gaps in functional genomic studies and water dropwort’s breeding.

The usability of transcriptomic data promotes the progress and development of reverse genetics research [2]. Virus-induced gene silencing (VIGS) has been extensively used to characterize gene functions in various plant species [13–15]. Viral infections activate a mechanism that defends against the viral RNA in host plants. This system is exploited by VIGS, which utilises posttranscriptional gene silencing (PTGS) that occurs when a virus infects host cells [16]. By inserting a part of the gene sequence into the virus, VIGS is a mechanism that can specifically target the mRNA of interest to initiate viral defence. When the recombinant virus replicates, double-stranded RNA (dsRNA) intermediates are generated, which activate RNA-mediated antiviral defence mechanisms that result in the formation of short interfering RNAs (siRNAs). These siRNAs then target the RNase complex to the corresponding RNA, ultimately leading to the disruption of the protein encoded by that RNA. [17]. The versatility of VIGS technology has made it an essential tool in simplifying the application of plant virus vectors in various plant species. Recently, some plant RNA or DNA viruses including, tobacco mosaic virus (TMV) [18], potato virus X (PVX) [19], tobacco rattle virus (TRV) [13, 20], barley stripe mosaic virus (BSMV) [21], tobacco mosaic virus satellite (TMVS) [22], pea early browning virus (PEBV) [23], tomato mosaic virus (ToMV) [24], bean pod mottle virus (BPMV) [25], cucumber mosaic virus (CMV) [26], brome mosaic virus (BMV) [27, 28], citrus tatter leaf virus (CTLV), apple latent spherical virus (ALSV) [29], tobacco ringspot virus (TRSV) [30], and rice tungro bacilliform virus (RTBV) [31], have been identified.

CMV is a typical member of the genus *Cucumovirus* of the family *Bromoviridae*. It is known to infect more than 1200 host species in over 100 plant families, including water dropwort [32]. CMV consists of three positively sensed single-stranded genomic RNAs (RNA1, 2, and 3) that express five proteins [33]. CMV express a total of five proteins, with RNA1 encoding the 1a protein and RNA2 encoding the 2a protein, both of which are responsible for viral genome replication [34]. RNA2 also includes the 2b gene, which encodes for the suppressor of RNA silencing [33, 35], a protein ultimately impairing protein activities associated with Argonaute 1 and Argonaute 4 by direct interaction [12, 25, 36–38]. Previous research studies suggest that the absence of a 2b gene results in CMV infecting plants systemically with only mild symptoms [39]. Furthermore, the 3a movement and coat proteins were encoded by RNA3, both of which play crucial roles in viral packaging and systemic movement. CMV is a prominent plant virus that is responsible for numerous plant diseases worldwide, while also serving as a pivotal model in studying plant-virus interactions. CMV has been successfully deployed as a VIGS agent in dicotyledonous plants such as *Nicotiana benthamiana* [26], *Glycine max* [40]*, Antirrhinum majus* [41]*, Solanum lycopersicum*, and *Capsicum annuum* [42], as well as monocotyledonous maize; whether CMV can induce silencing in water dropwort remains unknown.

We modified CMV-Fny into pCB301 vector. It was used to reduce the expression of the *PDS* and *GID1* genes in water dropwort and *N. benthamiana*. Following this, we proceeded to introduce CMV-Fny into the VbMS vector in order to explore the functional significance of two distinct miRNAs in both water dropwort and *N. benthamiana*. Through our experimentation, we demonstrated that the CMV-VIGS vector serves as a highly effective and efficient solution for conducting functional genomic research pertaining to water dropwort.

## Results

### Construction of CMV VIGS vector

**Fig. 1:**
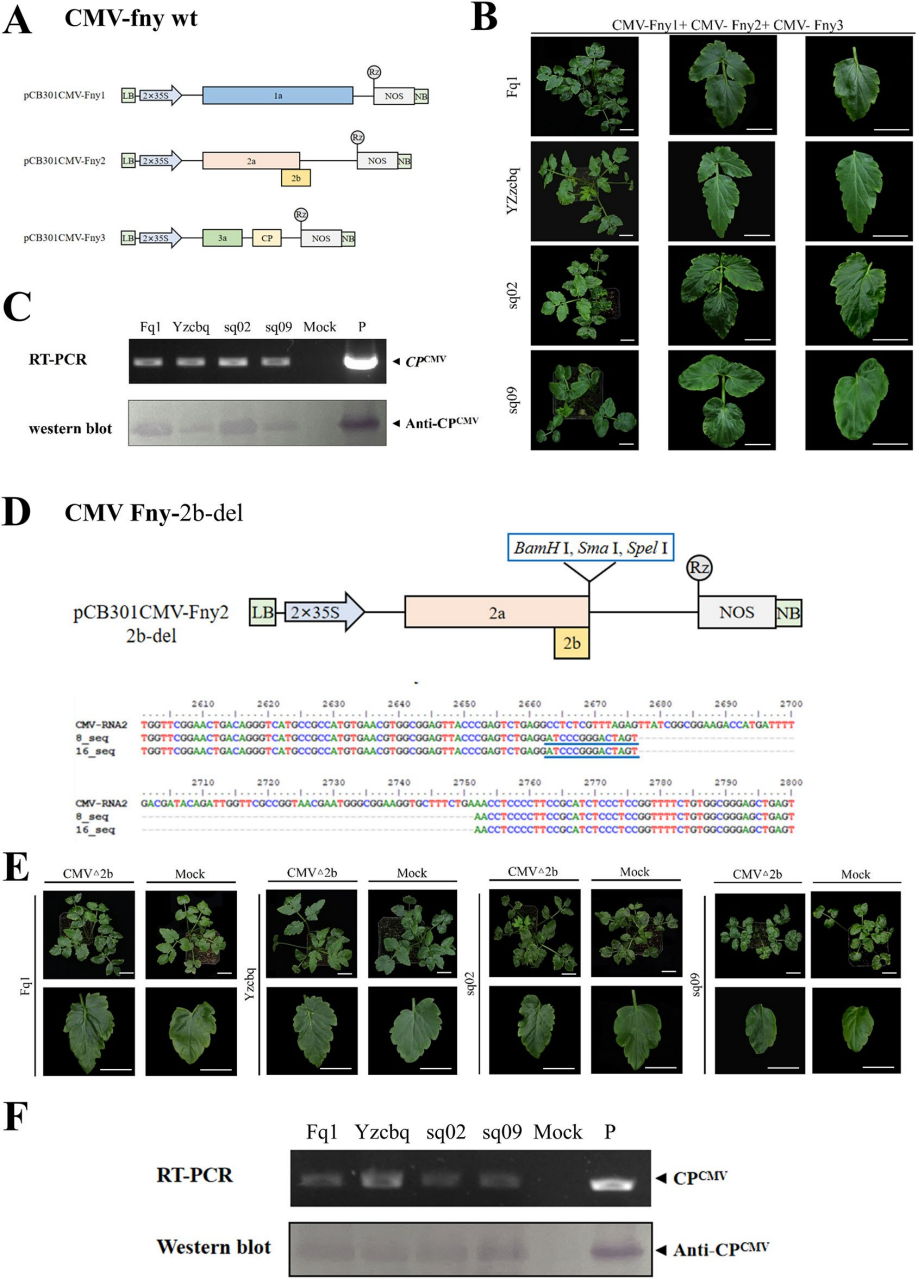
Construction of the CMV VIGS vector. **A**, Construct map of CMV-Fny. **B**, Symptoms of CMV-Fny in *O. javanica* (4 wpi). Scale bars are 2 cm. **C**, Detection of CMV-Fny-infected *O. javanica* by agro-infection (4 wpi). The vector plasmid with the CMV RNA3 insert was amplified as the positive (lane P) control. **D**, Construct map of the CMV-Fny 2b deletion mutant. **E**, Symptoms of the CMV-Fny 2b deletion mutant in *O. javanica*. Scale bars are 2 cm. **F**, Detection of CMV-Fny 2b deletion mutant-infected *O. javanica* by agro-infection (4 wpi). The vector plasmid with the CMV RNA3 insert was amplified as the positive (lane P) control

To analyse the suitability of the cucumber mosaic virus Fast New York strain (CMV-Fny) to induce gene silencing, the three chains of CMV-Fny from Prof. Xianbing Wang's laboratory were initially transferred into the pCB301 vector to build pCB301CMV-Fny1, pCB301CMV-Fny2, and pCB301CMV-Fny3 (Fig. 1A). The three *Agrobacterium tumefaciens* strains, individually harbouring the three different pCB301CMV-Fny constructs, were used together to infect four varieties of water dropwort, including ‘Fq1’, ‘Yzcbq’, ‘sq02’ and ‘sq09’. Mild shrinking appeared in the non-inoculated upper (that is, systemic) leaves at four weeks post agroinfiltration (wpi) (Fig. 1B). In addition to symptom observation, we extracted RNA and total protein from its systemic leaves to prepare samples; the utilization of both RT-PCR and western blotting techniques identified the presence of CMV in plant specimens that had been deliberately inoculated with CMV-Fny, thereby establishing the occurrence of CMV infection. (Fig. 1C).

In order to create a gene silencing vector for CMV, a modified form of pCB301CMV-Fny2 was utilized. The vector was customized by the addition of restriction sites that facilitated the cloning of a sequence. The RNA 2 consists of two ORFs, namely ORF 2a and 2b. It is noteworthy that the 2b ORF is integral for CMV symptom development, yet it is dispensable for viral replication and systemic infection. Fny 2b del BamSmaSpe-F and Fny 2b del Bam-R were used as primers for reverse PCR amplification to delete the nonoverlapping region of ORF 2b [43]. We introduced new *BamH* I*, Sma* I, and *Spe* I recognition sites (Fig. 1D, Additional file 1: Fig. S1). The 2b deletion mutant pCB301-Fny2-2b del was used to complete the transformation of the CMV infection clone. In addition, *A. tumefaciens* cells harbouring pCB301CMV-Fny1, pCB301CMV-Fny3 and pCB301-Fny2-2b del were infiltrated into leaves of *N. benthamiana* and *O. javanica.* Since infected plants showed no obvious symptoms, the treated plant leaves were used to prepare samples, and the target bands’ CMV-CP were detected by RT-PCR and western blotting (Fig. 1E and F; Additional file 1: Fig. S2). The results indicated that the modified CMV VIGS vector could be used in subsequent studies.

### Development of CMV-Fny as a virus-induced gene silencing (VIGS) vector

To build upon the accomplishments of the current inoculation method, we sought to investigate the CMV VIGS vector in promoting gene silencing within both *N. benthamiana* and water dropwort. A 332 bp fragment from the C-terminus of the *PDS* gene, necessary for carotenoid pigment production, was inserted into the CMV-Fny 2b deletion mutant (Additional file 1: S3A and B). CMV-Fny1 agro-infection, CMV-Fny3 agro-infection, and CMV-Fny2-2b-*NbPDS* agro-infection were infiltrated into *N. benthamiana* leaves and assayed for phenotypes and silencing efficacy. *N. benthamiana* that were subjected to recombinant CMV-*NbPDS* experienced the phenotypes of photobleaching on their leaves, with visible effects observed after 14 days of inoculation (Additional file 1: Fig. S3C). Total RNA and protein extracts extracted from Mock, CMVΔ2b, CMV-*NbPDS*, and CMV agroinfiltrated plants at 14 dpi were used for the detection of CMV by RT-PCR and western blotting (Additional file 1: Fig. S3D). CMV infection was confirmed in all CMVΔ2b, CMV-*NbPDS*, and CMV agroinfiltrated plants. In addition, the expression of *PDS* in CMV-*NbPDS*-infected plants decreased significantly compared to CMVΔ2b and CMV agroinfiltrated plants by semiquantitative RT-PCR (Additional file 1: Fig. S3D). The silencing efficiency was 70%–90% in *N. benthamiana* plants (Additional file 2: Table S1). Overall, these results demonstrate that the CMV VIGS vector can be used to silence the *NbPDS* gene.

**Fig. 2:**
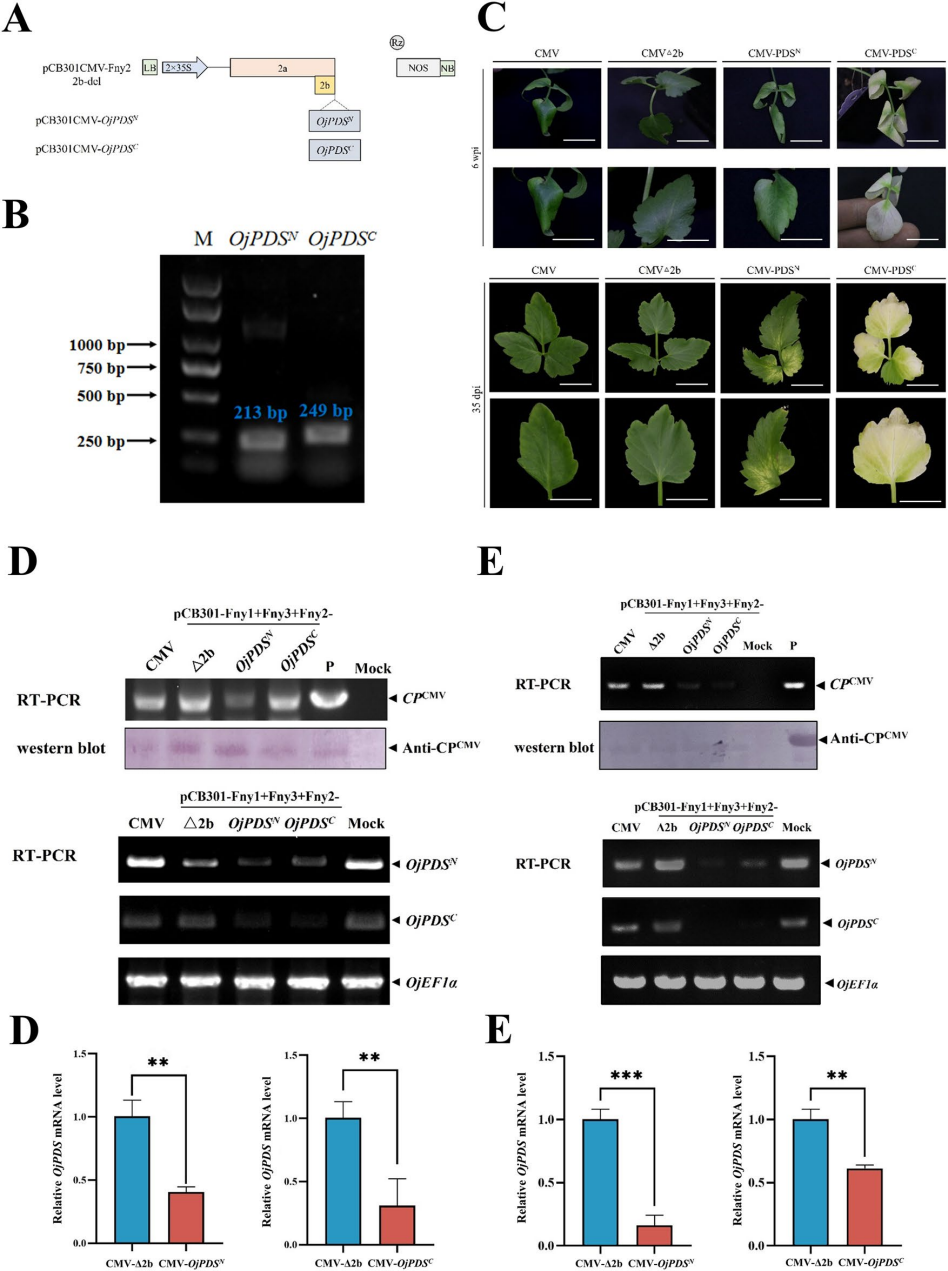
Silencing of the *OjPDS* gene in *O. javanica* using the CMV VIGS vector. **A**, Construct diagram of infectious clones of pCB301CMV-*OjPDS.* **B**, RT‒PCR detection of the *PDS*^*N*^ and *PDS*^*C*^ of *O. javanica.* **C**, Phenotypes of pCB301CMV-*OjPDS* in *O. javanica* (4 wpi). Scale bars are 2 cm. **D**, Detection of pCB301CMV-*OjPDS*-infected ‘Fq1’ by agro-infection (4 wpi). The vector plasmid with the CMV RNA3 insert was amplified as the positive (lane P) control. **E**, Detection of pCB301CMV-*OjPDS*-infected ‘Yzcbq’ by agro-infection (4 wpi). The vector plasmid with the CMV RNA3 insert was amplified as the positive (lane P) control

Next, we tested whether the CMV VIGS vector is suitable for gene silencing in water dropwort. The *PDS* gene of water dropwort was identified through the transcriptome data of water dropwort, while phylogenetic analysis revealed that it had the closest genetic relationship with the *Daucus carota PDS* gene (Additional file 1: Fig. S4A). In this regard, the conservative sequences of the N-terminus and C-terminus of the *OjPDS* gene were amplified to obtain 213 bp and 249 bp fragments that were inserted into the CMV-Fny 2b deletion mutant (Fig. 2A; Additional file 1: Fig. S4). Convert the recombinant virus into *Agrobacterium* competent cell and infiltrated water dropwort leaves via *Agrobacterium* infiltration. The newly born leaves of ‘Yzcbq’, inoculated with recombinant pCB301CMV-*OjPDS*^*N*^ and pCB301CMV-*OjPDS*^*C*^*,* showed obvious symptoms of photobleaching after 35 days of inoculation (Fig. 2C). The systematically infected leaves in 'Fq1' also became white after six weeks (Fig. 2C). The positive bands detected in agroinfiltrated plants were consistent with the size of the target band by RT-PCR and western blotting (Fig. 2D and E). Semiquantitative PCR and RT-qPCR showed that the expression levels of *OjPDS*^*N*^ and *OjPDS*^*C*^*,* after silencing, were significantly lowered than those of the control (Fig. 2D and E). The silencing efficiency reached 37.5%–75% in ‘Fq1’ and ‘Yzcbq’ plants (Additional file 2: Table S2). The expression levels of both, *PDS*^*N*^ and *PDS*^*C*^*,* were detected in 'Fq1' and 'Yzcbq' silencing *PDS* genes (Additional file 2: Tables S3 and S4). The successful silencing of *PDS* genes by the CMV VIGS vector thus indicated that the CMV VIGS vector of water dropwort can study the gene function of water dropwort.

### Silencing of *GID1* genes affected the growth and development of water dropwort

**Fig. 3:**
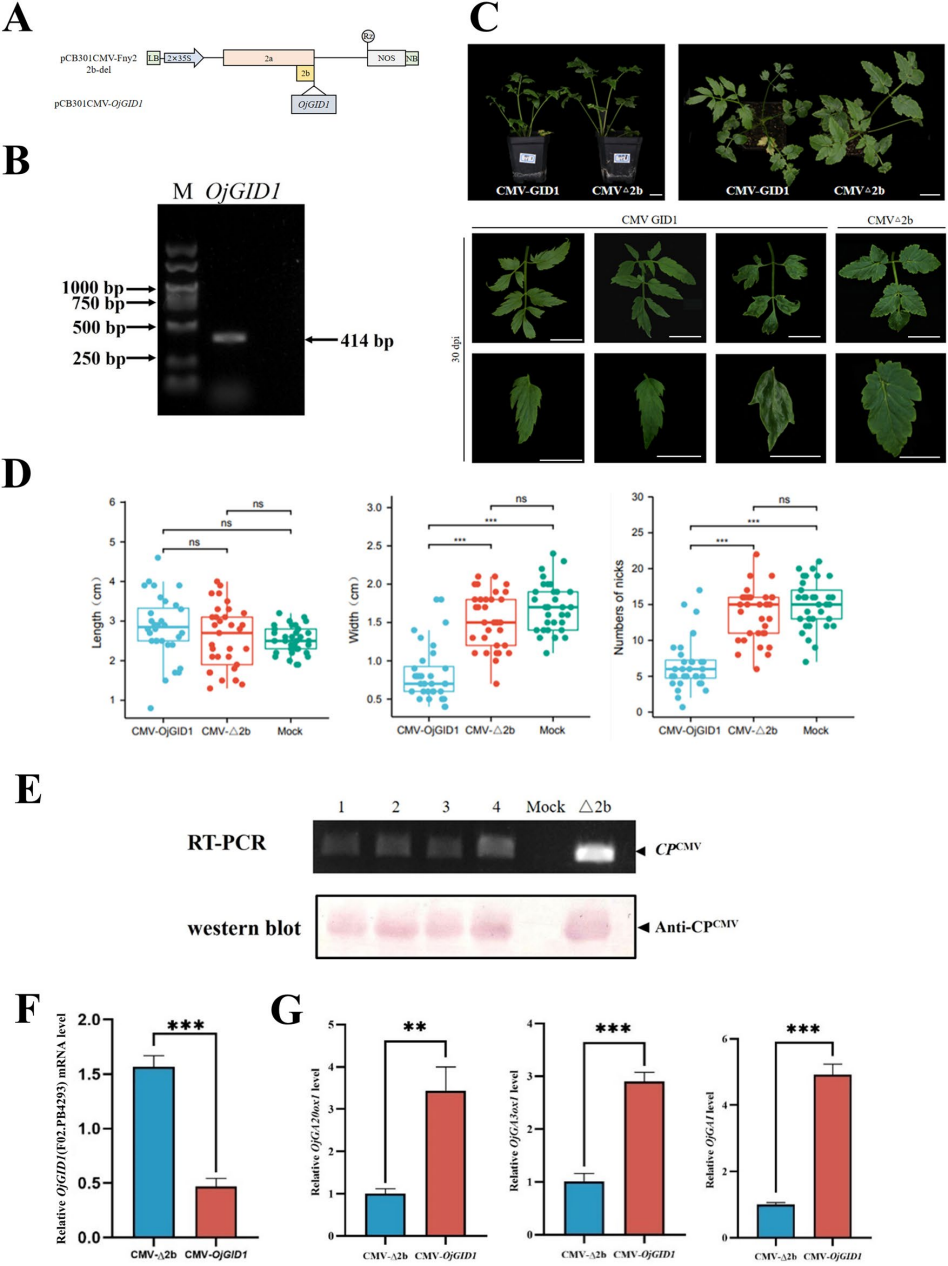
Silencing of the *OjGID1* gene in *O. javanica* using the CMV VIGS vector. **A**, Construct diagram of infectious clones of pCB301CMV-*OjGID1*; **B**, RT‒PCR detection of *GID1* of ‘Yzcbq’. **C**, Phenotypes of pCB301CMV-*OjGID1* in ‘Yzcbq’ (30 dpi). Scale bars are 2 cm. **D**, Systematic leaf morphology analysis of pCB301CMV-*OjGID1* in ‘Yzcbq’ (30 dpi). **E** Detection of pCB301CMV-*OjGID1*-infected *O. javanica* by agro-infection (30 dpi). **F** RT-qPCR analysis of *OjGID1* expression levels in CMV△2b- or CMV-*OjGID1*-infected plants. **G** RT-qPCR analysis of GA-related genes expression levels in CMV△2b- or CMV-*OjGID1*-infected plants

To further assess the ability of the CMV VIGS vector to silence endogenous genes in water dropwort, we investigated the roles of *GID1* in the development of water dropwort. According to the transcriptome data of water dropwort, a total of seven *OjGID1* gene sequences were found through phylogenetic analysis, closely related to the *GID1* gene of *D. carota* and *Coriandrum sativum* (Additional file 1: Fig. S5A). The conserved region found by sequence alignment was amplified a fragment of size 414 bp (Fig. 3B; Additional file 1: Fig. S5B and S5C). The obtained fragment was inserted into the CMV-Fny 2b deletion mutant to construct the pCB301CMV-*OjGID1* vector (Fig. 3A). Similarly, it was transformed into the *Agrobacterium* competent cell and injected into the water dropwort plant via *Agrobacterium* infiltration. After being infected with CMV-*GID1*, compared to the plants agroinfiltrated with CMV-Δ2b, 'Yzcbq' exhibited considerable phenotypic alterations by the 30th day post-infection. The CMV-*GID1*-inoculated plants were notably shorter in height and had longer and thinner leaves. (Fig. 3C). Statistical analysis of systematic leaf morphology showed that after silencing the *OjGID1* gene, leaf length changed insignificantly, but the leaf width and the number of nicks were significantly lowered than those of the control (Fig. 3D). CMV infection was confirmed in agroinfiltrated plants by RT-PCR (Fig. 3E). RT-qPCR analyses confirmed that the expression of *OjGID1* was significantly downregulated in water dropwort (Fig. 3F; Additional file 1: Fig. S6D and S7). The expression of *GA20ox1*, *GA3ox1*, and *GAI* as signaling pathway-related genes were upregulated by RT-qPCR (Fig. 3G). In addition, CMV-*GID1*-infected ‘Fq1’ plants showed the same silencing phenotypes (Additional file 1: Fig. S6A). The silencing efficiency was 37.5%–62.5% in ‘Fq1' and 'Yzcbq' plants (Additional file 2: Table S5). A similar phenotype was observed in *N. benthamiana*, where the silencing efficiency reached 50–75% (Additional file 1: Fig. S8; Additional file 2: Table S5). Overall, silencing *OjGID1* had dramatic effects on the growth and development of water dropwort and *N. benthamiana* plants.

### Silencing of water dropwort miRNA by the CMV VIGS vector

**Fig. 4:**
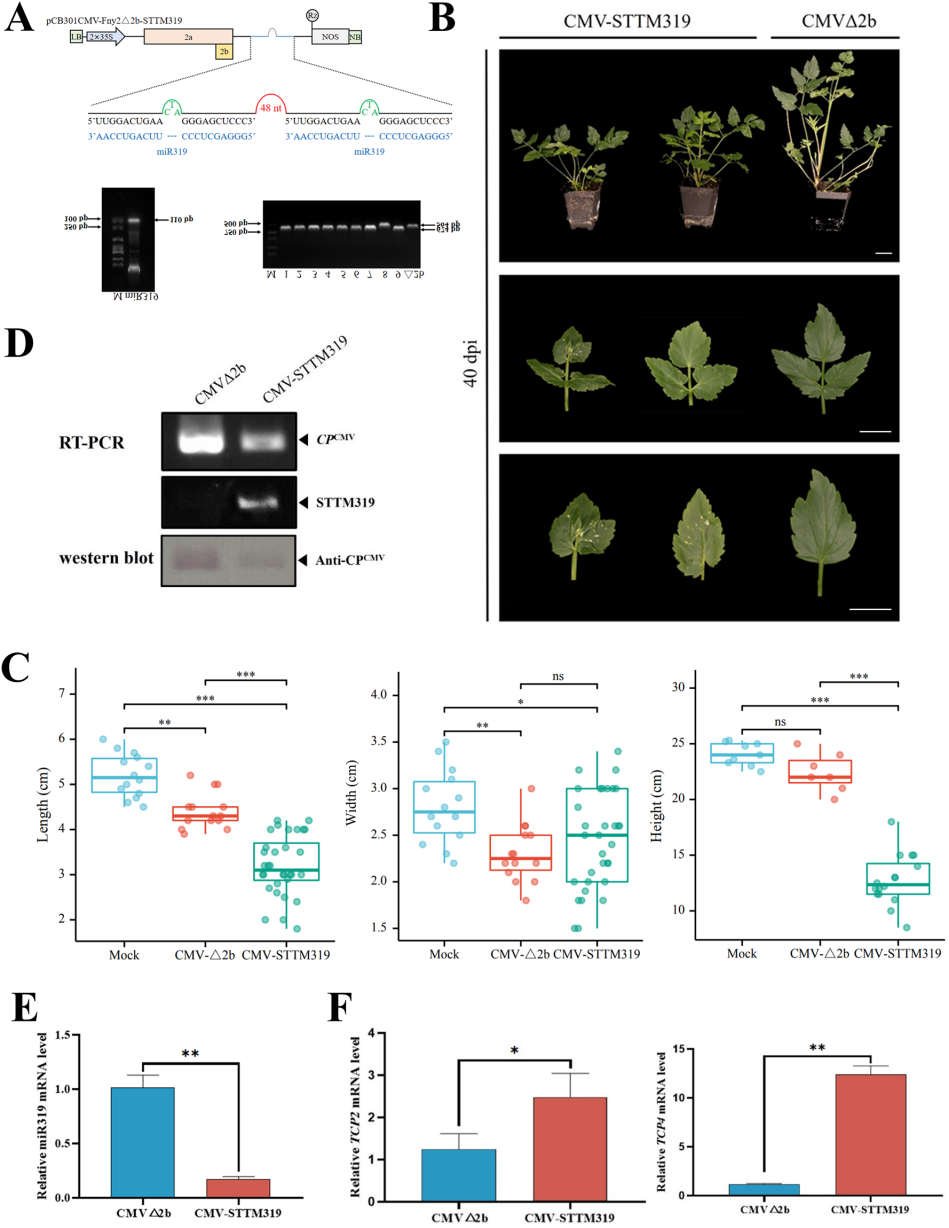
Silencing of miR319 in *O. javanica* using the CMV-based VbMS vector. **A**, Schematic representation of VbMS of miR319. **B**, Phenotypes of plants inoculated with CMV-STTM319 at 40 dpi. **C**, Systematic leaf morphology analysis of pCB301CMV-STTM319 in ‘Fq1’ (40 dpi). Scale bars are 2 cm. **D**, RT-PCR detection confirmed the expression of STTM319 in CMV-STTM319-infected plants. **E**, Stem-loop RT-qPCR analysis of miR319 expression levels in CMV△2b- or CMV-STTM319-infected plants. **F**, Detection of the relative expression levels of the miR319 target genes *TCP2* and *TCP4*

We studied the silencing of water dropwort miRNA by the CMV VIGS vector to further explore the latter’s application in water dropwort. Previous studies on miRNA of Apiaceae plants and celery have shown that miR319 regulated four *TCP* family genes in celery, four in coriander, and three in carrot [44, 45]. The target genes’ *TCP2* and *TCP4* sequences for water dropwort were obtained by blasting *TCP2* and *TCP4* sequences in carrot and celery to the transcriptomic data of water dropwort. The well-characterized miR319 was cloned into the pCB301CMV-Fny2-2b-del vector using a short tandem target mimic (STTM) strategy (Fig. 4A). By 40 dpi, CMV-STTM319-infected ‘Fq1’ plants showed phenotypes in which the plants exhibited a dwarf phenotype, and the leaves were significantly smaller than the control (Fig. 4B). Statistical analysis of systematic leaf morphology showed that after silencing STTM319, the leaf length, width, and height were significantly lowered than those of the control (Fig. 4C). The STTM319 expression in the CMV-STTM319-infected plants was detected by RT-PCR (Fig. 4D). Additionally, the findings of RT-qPCR detection revealed that in the plants infected with CMV-STTM319, the miR319 level was dramatically decreased while the target miRNA level increased (Fig. 4E and F). CMV-STTM319-infected ‘Yzcbq’ plants showed the same silencing phenotypes (Additional file 1: Fig. S9). The silencing efficiency was 37.5%–75% in ‘Fq1' and 'Yzcbq' plants (Additional file 2: Table S6). A similar phenotype was observed in *N. benthamiana*, in which the silencing efficiency reached 50–62.5% (Additional file 1: Fig. S10; Additional file 2: Table S6).

**Fig. 5:**
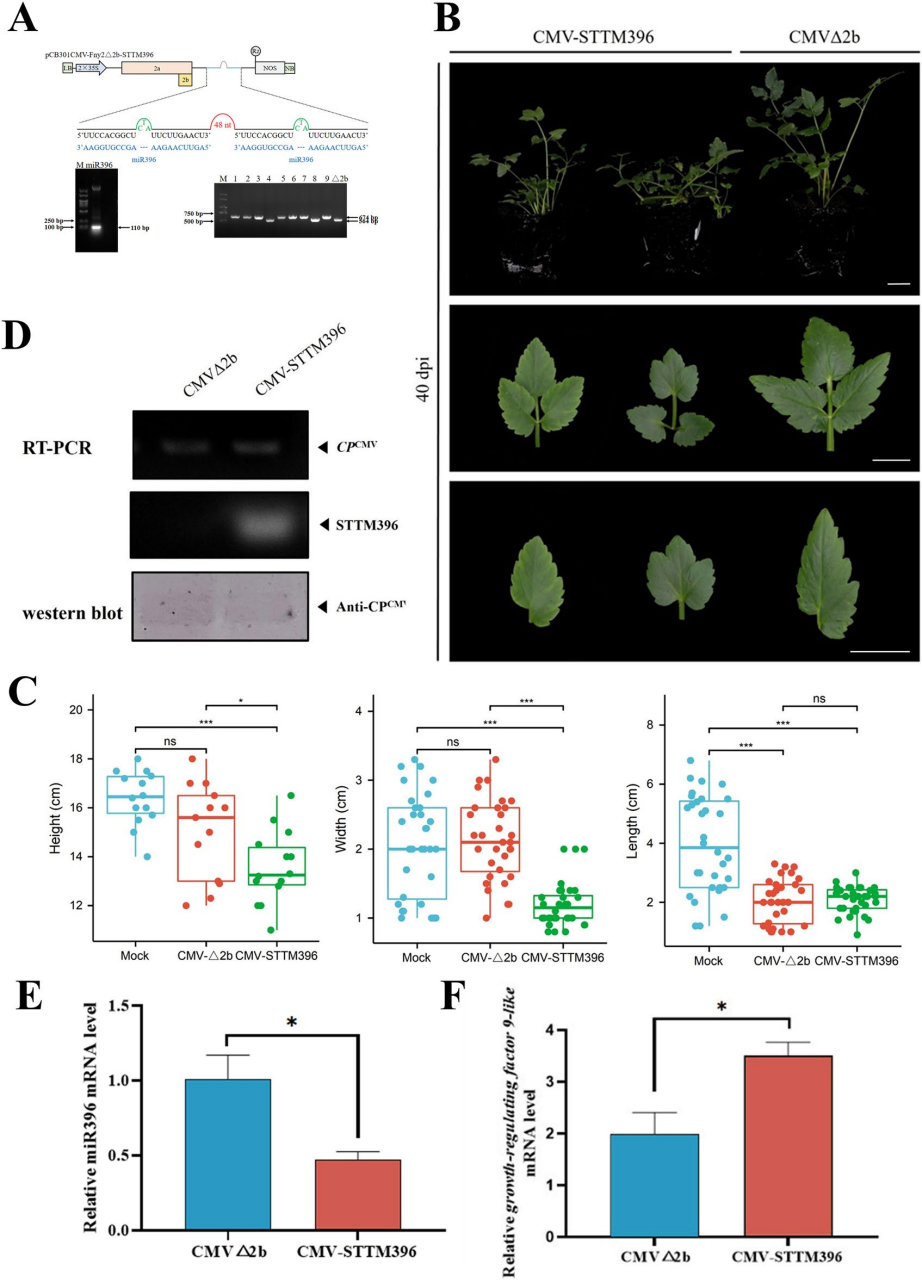
Silencing of miR396 in *O. javanica* using the CMV-based VbMS vector. **A**, Schematic representation of VbMS of miR396. **B**, Phenotypes of plants inoculated with CMV-STTM396 at 40 dpi. **C**, Systematic leaf morphology analysis of pCB301CMV-STTM396 in ‘Fq1’ (40 dpi). Scale bars are 2 cm. **D**, RT‒PCR detection confirmed the expression of STTM396 in CMV-STTM396-infected plants. **E**, Stem‒loop RT‒qPCR analysis of miR396 expression levels in CMV△2b- or CMV-STTM396-infected plants. **F**, Detection of the relative expression level of the miR396 target gene *Growth-Regulating Factor 9-like*

The miR396 sequence and target gene *Growth-Regulating Factor 9-like* sequences of water dropwort were obtained by blasting *Growth-Regulating Factor 9-like* sequences in celery to the transcriptomic data of water dropwort [45]. The well-characterized miR396 was cloned into the pCB301CMV-Fny2-2b-del vector using an STTM strategy (Fig. 5A). By 40 dpi, CMV-STTM396-infected ‘Fq1’ plants showed a silencing phenotype in which the plants exhibited a dwarf phenotype (Fig. 5B). Statistical analysis of systematic leaf morphology showed that after silencing STTM396, the leaf length, width, and height were significantly lowered than those of the control (Fig. 5C). RT-PCR results showed that STTM396 was normally expressed in CMV-STTM396-infected water dropwort plants (Fig. 5D). In addition, results of fluorescence quantitative detection showed that the expression of miR396 was increased (Fig. 5E), while that of *Growth-Regulating Factor 9-like*, which is a target gene of miR396, was significantly lowered than that of the control (Fig. 5F). CMV-STTM396-infected ‘Yzcbq’ plants showed the same silencing phenotypes (Additional file 1: Fig. S11). The silencing efficiency was 37.5%–62.5% in ‘Fq1' and 'Yzcbq' plants (Additional file 2: Table S7). A similar phenotype was observed in *N. benthamiana*, in which the silencing efficiency reached 50–75% (Additional file 1: Fig. S12; Additional file 2: Table S7). Based on the findings, it appears that the CMV VbMS technique has the ability to suppress miRNAs and offer valuable insights into the roles of miRNA and target mRNA interactions.

## Discussion

At present, the genetic transformation, gene silencing, and gene knockout systems for water dropwort have not been well established, with no corresponding technical support for gene function research. VIGS offers an alternate method for examining how plant genes work. We created a CMV-based gene silencing vector to address this problem. Based on the CMV-Fny infectious clone, the 2b deletion mutant vector, pCB301-Fny2-2b del, was constructed to transform the CMV-Fny infectious clone of water dropwort (Fig. 1D). Otagaki et al. (2016), for the first time, carried out the deletion mutation of CMV 2b protein, successfully constructed the CMV2-A1 vector, and proved that it was suitable for the rapid induction of transcription and posttranscriptional gene silencing [26]. Hong et al. successfully silenced the *PDS* gene in tomatoes and peppers. In this research, the test method was used to perform deletion mutations of amino acids 81 ~ 111 of the CMV 2b protein through reverse PCR amplification, to obtain its deletion mutant [42]. Based on pCMV201, four VIGS vectors expressing 2b proteins of different lengths namely, ZMBJ-CMV-2b, ZMBJ-CMV-2b_N94_, ZMBJ-CMV-2b_N81_ and ZMBJ-CMV-2b_N61_, were constructed [46]. Tzean et al., in their study, inserted the restriction site at the end of the CMV 2a protein and constructed the CMV-VIGS vector in banana by deletion of the CMV 2b protein [47]. By two distinct strains, CMV-ZMBJ and CMV-Fny, Li et al. (2021) generated a pseudo recombinant-chimeric (Pr) CMV [48]. By employing a ligation-independent cloning (LIC) technique, they devised an altered Pr CMV-LIC VIGS vector, which facilitates straightforward gene cloning for carrying out extensive silencing in maize. In addition, when the modified CMV VIGS vector was inoculated into *N. benthamiana*, the upper leaves showed mild symptoms of shrinkage in the early stages and disappeared in the middle and late stages, which was consistent with the test results of Yao Min [43] (Additional file 1: Fig. S2). Although 2b deletion did not fully manifest disease in the early stages, it significantly affected the symptoms brought on by the virus in *N. benthamiana*, suggesting that the 2b protein is crucial for *N. benthamiana*'s CMV symptoms. The specific mechanism causing this phenomenon needs to be further explored.

The *PDS* gene is utilized to produce visual phenotypes in plant VIGS experiments for various species, which are involved in carotenoid biosynthesis. In water dropwort, silencing *OjPDS*^*N*^ and *OjPDS*^*C*^ with the CMV-based VIGS system led to photobleaching that affected both newly developed leaves and stems (Fig. 2; Additional file 1: Fig. S3). This demonstrates the highly efficient nature of the CMV-based VIGS system in quickly silencing genes in water dropwort. Herefore, gene silencing approach has the potential to be extremely beneficial in carrying out functional investigations related to the developmental and biosynthetic pathways of water dropwort genes.

The gibberellin signaling pathway heavily relies on the Gibberellin receptor *GID1*. GA is a crucial hormone responsible for plant growth and frequently resulted in dwarfing. To analyze the *GID1* gene that encodes GA receptors, specifically *OjGID1*, we extracted it from the water dropwort genome. VIGS was performed to silence the *OjGID1* gene, which led to a dwarfish appearance (Fig. 3; Additional file 1: Fig. S6). Additionally, the *OjGID1* gene silencing had a significant impact on papaya leaf morphology [49]. Our experiment revealed that CMV-*OjGID1* infection triggered *GID1* silencing phenotypes comparable to those in tobacco rattle virus-induced gene silencing of *GID1* in petunia and *Arabidopsis thaliana* and tomato *GID1* mutants [50–52]. Hence, our study provides a sound foundation for future plant breeding and production.

As well as engineering CMV for utilization as a VIGS vector, we also its miRNA silencing function. It represents the initial VbMS vector designed for the functional assessment of miRNAs relating to water dropwort. Amongst the miRNA families, miR319 is highly conserved and integral in leaf development, it regulates the expression of a multitude of genes, including teosinte branched1, cycloidea, and proliferating cell nuclear antigen binding factor (*TCP*) genes [53]. In this study, ‘Yzcbq’ and ‘Fq1’ were selected as the experimental materials. This variety showed obvious stem elongation characteristics during the growth of water dropwort, which were conducive to the observation of phenotype. The results show that CMV VIGS system expressed the STTM319. After 30 days of inoculation, it was observed that the leaf morphology was significantly smaller than that of the control, and the entire plant showed an obvious dwarf phenotype (Figs. 4B and 5B; Additional file 1: Fig. S9 and S10). Fang expressed STTM319 through the TRSV silencing system; after 30 days of watermelon inoculation, it was discovered that both the plant and leaf size were noticeably shortened. These results were in agreement with the phenotypic observations from the study conducted, as previously established in [54]. Additionally, the experimental findings indicated a crucial outcome, whereby the expression level of the target gene *TCP4*, demonstrated a substantial increase on miR319 silencing. Significantly, this finding was found to be congruent with the results obtained from the watermelon STTM319 analysis (Fig. 4F; Additional file 1: Fig. S8E and S9E). The ectopic expression of *Arabidopsis* miR319-resistant *TCP4* (*mTCP4*) has been found to lead to the production of micro leaves [55]. Moreover, Jones-Rhoades and Bartel discovered miR396 in *Arabidopsis* and rice in 2004 and predicted that it can regulate the *Growth-Regulating Factor* (GRF) gene [56]. In this study, STTM396 was silenced by CMV VIGS. miR396-silenced water dropwort plants were found to be shorter than control plants 30 days after inoculation (Fig. 5B; Additional file 1: Fig. S11A and S12A). Liu et al. found that the transgenic *A. thaliana* overexpressing miR396 was significantly shorter than the control, which was consistent with the results of this experiment [57]. Therefore, the CMV silencing system established can be used for miRNA silencing tests. *Arabidopsis* miR396 has been reported to be able to target seven members of the GRF family and regulate leaf growth and shape [58, 59]. Jia measured the expression level of miR396 in water dropwort leaves at three different developmental stages and found that it showed a gradual downwards trend, indicating that miR396 was involved in the development of celery leaves [45]. Similarly, the leaf size of miR396 silenced water dropwort was smaller than that of the control, which was consistent with the experimental results by Jia [45] (Fig. 5B; Additional file 1: Fig. S11A and S12A).

According to the findings of the research, CMV-based vectors demonstrate a remarkable capacity for exploring the biological roles of genes and miRNAs in water dropwort. Considering the versatility of vectors, it is evident that functional genomic studies in water dropwort plants are likely to be significantly facilitated and wildly used. The VIGS systerm plays a good role in silencing the expression of *PDS*, *G1D1* and miRNAs in water dropwort for subsequent studies. In our experiments, water dropwort subjected to VIGS still exhibited silencing after 60–90 dpi (data not shown). The miRNA319 silencing efficiency was also superior to previous study [30]. The tissue cultured seedlings can effectively improve the consistency and efficiency of the results. Temperature and the length of insertion fragments are essential to large-scale and efficient application of VIGS in the gene function-related validation experiments. Therefore, there is great potential for these vectors to reveal more about the molecular mechanisms of water dropwort plants and establish a theoretical foundation for future studies of this species.

## Materials and methods

### Plant materials

The study utilized various types of water dropwort buds. The plants subjected to VIGS assays were cultivated in a greenhouse, with a temperature of 25 °C, and exposed to a light/dark cycle of 10/14 h. Moreover, the plants were kept in an environment with relative humidity at 85%.

### Vector construction

*Agrobacterium*-mediated infectious cDNA clones of CMV-Fny were obtained from Prof. Dawei Li's laboratory. The three chains of CMV-Fny were transferred into the mini binary vector, pCB301, to build pCB301CMV-Fny1, pCB301CMV-Fny2, and pCB301CMV-Fny3, which were then introduced into *Agrobacterium* and inoculated into *N. benthamiana* and water dropwort by agroinfiltration. pCB301CMV-Fny2 was used as the DNA template; Fny 2b del BamSmaSpe-F and Fny 2b del Bam-R were used as primers for reverse PCR amplification, and a fragment of approximately 7 566 bp size was obtained [43] (Additional file 1: Fig. S1). The PCR product was recovered, the 2b deletion mutant vector pCB301-Fny3-2b del was constructed by *BamH* I enzyme digestion and self-linking, and the modification of the CMV-infected clone of water dropwort was completed. It indicated that the modified CMV VIGS vector could be used in subsequent studies.

### Agroinfiltration of *N. benthamiana* and water dropwort plants

The plasmids were transformed into individual *A. tumefaciens* GV3101 cells [60]. The cells were cultured at 28 °C with shaking at 220 rpm for 10–16 h. After centrifuging the cells at 3000 × g for 10 min, they were suspended in an infiltration buffer [61]. The cultures containing the construct of CMV infectious clone were adjusted to an optical density of OD_600_ = 0.6, and then incubated at room temperature for 2–3 h. *N. benthamiana* and water dropwort plants were injected with the *Agrobacterium* cultures using a sterile 1.0 mL syringe.

### RNA extraction and RT‒PCR

The MiniBEST Plant RNA Extraction Kit (polysaccharide polyphenol Plant tissue lysis) manufactured by TAKARA in Dalian was utilized to extract total RNA from leaf tissues of *N. benthamiana* and water dropwort. The manufacturer’s standardized protocol was followed during the RNA extraction procedure. To determine RNA quality, a 1.0% agarose gel was utilized. Single-stranded cDNA was synthesized by the PrimeScript™ II 1st Strand cDNA Synthesis Kit, following the instructions provided by TAKARA, Dalian. The resulting cDNA solution was preserved at a temperature of − 20 °C.

The RT-PCR assay utilized specific primers that were listed in the Additional file 2: Table S10. The RT-PCR mixture had a volume of 25 μL and contained 0.2 μL of Ex Taq HS (5 U/μL) from TAKARA in Dalian, 2.0 μL of cDNA, 2.0 μL of dNTP (10 mM each), 2.5 μL of primers, 2.5 μL of 10 × PCR Buffer II, and the appropriate amount of ddH_2_O. Following its creation, a 5 μL sample was tested on a 1.0% agarose gel. All of the samples were then subjected to RT-PCR testing, and any positive samples. were stored at − 80 ℃.

### Protein extractions and western blotting

To extract total protein, the leaf tissue infected with CMV was frozen in liquid nitrogen and then ground to a fine powder. Next, the powder was suspended in a protein extraction buffer composed of 1 M Tris–HCl (pH 6.8), 200 mL of glycerin, 2 g of bromophenol blue, and 50 mL of β-mercaptoethanol. The resultant samples were boiled for 10 min, after which they were centrifuged at 12,000 rpm for 10 min. Supernatants were selected and subjected to SDS-PAGE on a vertical electrophoresis apparatus. Next, the membrane was blocked with gentle agitation in 10 mL of non-fat milk in TBST (20 mM Tris base, 150 mM NaCl, 0.05% tween-20, pH 7.5) added with the CP-specific antibody at 4 °C overnight. Following this, the membranes were washed thrice with a 1 × solution of TBST every 10 min. A coupled anti-rabbit immunoglobulin was then incubated at an appropriate working dilution for 45 min. The NC membranes were subsequently washed with TBST for 10 min each. Finally, the membrane was photographed.

### Supplementary Information

**Additional file 1: Figure S1.** Construction of pCB301-CMV-RNA2△2b. (A) Map of pCB301-RNA2 and pCB301-RNA2△2b. (B) Inverse-PCR detection of CMV-RNA2. **Figure S2.** The CMV-Fny 2b deletion mutant infected *N. benthamiana* by agro-infection. (A) Symptoms of the CMV-Fny 2b deletion mutant in *N. benthamiana*. Scale bars are 2 cm. (B) Detection of CMV-Fny 2b deletion mutant-infected *N. benthamiana* by agro-infection (8 dpi). The vector plasmid with the CMV RNA3 insert was amplified as the positive (lane P) control. **Figure S3.** Silencing of the *NbPDS* gene in *N. benthamiana* using the CMV VIGS vector. (A) Construct diagram of infectious clones of pCB301CMV-*NbPDS*. (B) RT‒PCR detection of the *PDS*^*C*^ of *N. benthamiana*. (C) Phenotypes of pCB301CMV-*NbPDS* in *N. benthamiana* (14 dpi). Scale bars are 2 cm. (D) Detection of pCB301CMV-*NbPDS*-infected *N. benthamiana* by agro-infection (14 dpi). (E) RT‒qPCR analysis of *NbPDS* expression levels in CMV△2b- or CMV-*NbPDS*-infected plants. **Figure S4.** Identification of *PDS* gene in *O. javanica*. (A) Phylogenetic analysis of *PDS* of *O. javanica*. (B) Map of *PDS* gene of *O. javanica*. (C) Silencing Sequence of *PDS* gene in *O. javanica*. **Figure S5.** Identification of *GID1* gene in *O. javanica*. (A) Phylogenetic analysis of *GID1* of *O. javanica*. (B) Map of *GID1* gene of *O. javanica*. (C) Silencing Sequence of *GID1* gene in *O. javanica*. **Figure S6.** Silencing of the *OjGID1* gene in *O. javanica *using the CMV VIGS vector. (A) Phenotypes of pCB301CMV-*OjGID1* in *O. javanica *(30 dpi). Scale bars are 2 cm. (B) Systematic leaf morphology analysis of pCB301CMV-*OjGID1* in ‘Fq1’ (30 dpi). (C) Detection of pCB301CMV-*OjGID1*-infected ‘Fq1’ by agro-infection (30 dpi). (D) RT‒qPCR analysis of *OjGID1* expression levels in CMV△2b- or CMV-*OjGID1*-infected plants. (E) RT‒qPCR analysis of GA-related genes expression in CMV△2b- or CMV-*OjGID1*-infected plants. **Figure S7. **RT‒qPCR analysis of 6 *OjGID1 *genes expression in CMV△2b- or CMV-*OjGID1*-infected *O. javanica*. (A) RT‒qPCR analysis of 6 *OjGID1 *genes expression in CMV△2b- or CMV-*OjGID1*-infected ‘Yzcbq’. (B) RT‒qPCR analysis of 6 *OjGID1 *genes expression in CMV△2b- or CMV-*OjGID1*-infected ‘Fq1’. **Figure S8.** Silencing of the *NbGID1* gene in *N. benthamiana* using the CMV VIGS vector. (A) Phenotypes of pCB301CMV-*NbGID1* in *N. benthamiana* (17 dpi). Scale bars are 2 cm. (B) Systematic leaf morphology analysis of pCB301CMV-*NbGID1* in *N. benthamiana* (17 dpi). (C) Detection of pCB301CMV-*NbGID1*-infected *N. benthamiana* by agro-infection. (D) RT‒qPCR analysis of *NbGID1* expression levels in CMV△2b- or CMV-*NbGID1*-infected plants. (E) RT‒qPCR analysis of *NbGID1* expression levels in CMV△2b- or CMV-*NbGID1*-infected plants. **Figure S9.** Silencing of miR319 in *O. javanica *using CMV-based VbMS vector. (A) Phenotypes of plants inoculated with CMV-STTM319 at 30 dpi. Scale bars are 2 cm. (B) Systematic leaf morphology analysis of pCB301CMV-STTM319 in ‘Yzcbq’. (C) Detection of pCB301CMV-STTM319-infected ‘Yzcbq’ by agro-infection. (D) Stem‒loop RT‒qPCR analysis of miR319 expression levels in CMV△2b- or CMV-STTM319-infected plants. (E) Detection of the relative expression levels of the miR319 target genes *TCP2* and *TCP4*. **Figure S10.** Silencing of miR319 in *N. benthamiana *using CMV-based VbMS vector. (A) Phenotypes of plants inoculated with CMV-STTM319 at 15 dpi. Scale bars are 2 cm. (B) Systematic leaf morphology analysis of pCB301CMV-STTM319 in *N. benthamiana*. (C) Detection of pCB301CMV-STTM319-infected *N. benthamiana* by agro-infection. (D) Stem‒loop RT‒qPCR analysis of miR319 expression levels in CMV△2b- or CMV-STTM319-infected plants. (E) Detection of the relative expression levels of the miR319 target genes *TCP2* and *TCP4*. **Figure S11.** Silencing of miR396 in *O. javanica *using CMV-based VbMS vector. (A) Phenotypes of plants inoculated with CMV-STTM396 at 30 dpi. Scale bars are 2 cm. (B) Systematic leaf morphology analysis of pCB301CMV-STTM396 in ‘Yzcbq’. (C) Detection of pCB301CMV-STTM396-infected ‘Yzcbq’ by agro-infection. (D) Stem‒loop RT‒qPCR analysis of miR396 expression levels in CMV△2b- or CMV-STTM396-infected plants. (E) Detection of the relative expression level of the miR396 target gene *Growth-Regulating Factor 9-like*. **Figure S12.** Silencing of miR396 in *N. benthamiana *using CMV-based VbMS vector. (A) Phenotypes of plants inoculated with CMV-STTM396 at 15 dpi. Scale bars are 2 cm. (B) Systematic leaf morphology analysis of pCB301CMV-STTM396 in *N. benthamiana*. (C) Detection of pCB301CMV-STTM396-infected *N. benthamiana* by agro-infection. (D) Stem‒loop RT‒qPCR analysis of miR396 expression levels in CMV△2b- or CMV-STTM396-infected plants. (E) Detection of the relative expression level of the miR396 target gene *polyamine oxidase 1*. **Figure S13.** Naturally infected of water dropwort by CMV. (A) Symptoms of CMV in *O. javanica*. (B) RT-PCR and western blot detection of the water dropwort samples.

**Additional file 2: Table S1.** The silencing efficiency of *PDS* genes in *N. benthamiana*. **Table S2.** The silencing efficiency of *PDS* genes in water dropwort. **Table S3.** RT‒qPCR analysis of *OjPDS* expression levels in CMV-*OjPDS*^*N*^- or CMV-*OjPDS*^*C*^-infected ‘Fq1’. **Table S4. **RT‒qPCR analysis of *OjPDS* expression levels in CMV-*OjPDS*^*N*^- or CMV-*OjPDS*^*C*^-infected ‘Yzcbq’. **Table S5.** The silencing efficiency of *GID1* genes. **Table S6. **The silencing efficiency of miRNA319 genes. **Table S7. **The silencing efficiency of miRNA396 genes. **Table S8. **Primers used in this study.

## Data Availability

Source data can be found in Supplementary Datasets. The data that support the finding of this Conflict of interests study are available from the corresponding authors upon request.
