# Supplementary material for: Cucumber mosaic virus-induced gene and microRNA silencing in water dropwort (Oenanthe javanica (Blume) DC)
Source: Plant Methods. 2024 Jan 11;20:6. doi: 10.1186/s13007-023-01129-4 (PMC10782793; doi:10.1186/s13007-023-01129-4)
Supplement: Supplementary file 1 — Additional file 1: Figure S1. Construction of pCB301-CMV-RNA2△2b. (A) Map of pCB301-RNA2 and pCB301-RNA2△2b. (B) Inverse-PCR detection of CMV-RNA2. Figure S2. The CMV-Fny 2b deletion mutant infected N. benthamiana by agro-infection. (A) Symptoms of the CMV-Fny 2b deletion mutant in N. benthamiana. Scale bars are 2 cm. (B) Detection of CMV-Fny 2b deletion mutant-infected N. benthamiana by agro-infection (8 dpi). The vector plasmid with the CMV RNA3 insert was amplified as the positive (lane P) control. Figure S3. Silencing of the NbPDS gene in N. benthamiana using the CMV VIGS vector. (A) Construct diagram of infectious clones of pCB301CMV-NbPDS. (B) RT‒PCR detection of the PDSC of N. benthamiana. (C) Phenotypes of pCB301CMV-NbPDS in N. benthamiana (14 dpi). Scale bars are 2 cm. (D) Detection of pCB301CMV-NbPDS-infected N. benthamiana by agro-infection (14 dpi). (E) RT‒qPCR analysis of NbPDS expression levels in CMV△2b- or CMV-NbPDS-infected plants. Figure S4. Identification of PDS gene in O. javanica. (A) Phylogenetic analysis of PDS of O. javanica. (B) Map of PDS gene of O. javanica. (C) Silencing Sequence of PDS gene in O. javanica. Figure S5. Identification of GID1 gene in O. javanica. (A) Phylogenetic analysis of GID1 of O. javanica. (B) Map of GID1 gene of O. javanica. (C) Silencing Sequence of GID1 gene in O. javanica. Figure S6. Silencing of the OjGID1 gene in O. javanica using the CMV VIGS vector. (A) Phenotypes of pCB301CMV-OjGID1 in O. javanica (30 dpi). Scale bars are 2 cm. (B) Systematic leaf morphology analysis of pCB301CMV-OjGID1 in ‘Fq1’ (30 dpi). (C) Detection of pCB301CMV-OjGID1-infected ‘Fq1’ by agro-infection (30 dpi). (D) RT‒qPCR analysis of OjGID1 expression levels in CMV△2b- or CMV-OjGID1-infected plants. (E) RT‒qPCR analysis of GA-related genes expression in CMV△2b- or CMV-OjGID1-infected plants. Figure S7. RT‒qPCR analysis of 6 OjGID1 genes expression in CMV△2b- or CMV-OjGID1-infected O. javanica. (A) RT‒qPCR analysis of 6 OjGID1 genes expres [file 13007_2023_1129_MOESM1_ESM.docx]

Supplemental Table S1. The silencing efficiency of *PDS* genes in *N. benthamiana.*

| Plants | No. of *PDS*  silenced/inoculated plants | | Efficiency of  silencing *PDS* (%) | |
| --- | --- | --- | --- | --- |
| *N. benthamiana* | | |  |  |
| Repetition 1 | 9/10 | | 90% | |
| Repetition 2 | 7/10 | | 70% | |
| Repetition 3 | 7/10 | 70% | |  |

Supplemental Table S2. The silencing efficiency of *PDS* genes in water dropwort.

| Plants | No. of *PDS^N^*  silenced/inoculated plants | Efficiency of  silencing *PDS^N^* (%) | No. of *PDS^C^*  silenced/inoculated plants | Efficiency of silencing *PDS^C^* (%) |
| --- | --- | --- | --- | --- |
| Fq1 |  | | | |
| Repetition 1 | 4/8 | 50% | 4/8 | 50% |
| Repetition 2 | 5/8 | 62.5% | 3/8 | 37.5% |
| Repetition 3 | 4/8 | 50% | 5/8 | 62.5% |
| Yzcbq |  | | | |
| Repetition 1 | 5/8 | 62.5% | 4/8 | 50% |
| Repetition 2 | 3/8 | 37.5% | 5/8 | 62.5% |
| Repetition 3 | 6/8 | 75% | 3/8 | 37.5% |

Supplemental Table S3. RT‒qPCR analysis of *OjPDS* expression levels in CMV-*OjPDS^N^*- or CMV-*OjPDS^C^*-infected ‘Fq1’.

| Plants | **CMV-*OjPDS^N^*** | | | | | | **CMV-*OjPDS^C^*** | | | | | |
| --- | --- | --- | --- | --- | --- | --- | --- | --- | --- | --- | --- | --- |
| **‘Fq1’-*OjPDS*** | 25.05 | 24.79 | 25.19 | 24.82 | 24.76 | 25.36 | 24.80 | 24.90 | 25.14 | 24.54 | 24.83 | 24.74 |
|  | 25.68 | 25.40 | 25.26 | 25.51 | 25.58 | 25.33 | 24.89 | 24.97 | 25.09 | 24.18 | 24.57 | 24.31 |
|  | 25.05 | 25.16 | 25.35 | 25.72 | 25.21 | 25.05 | 24.78 | 24.89 | 25.06 | 24.59 | 24.67 | 24.98 |
|  | 25.89 | 25.53 | 25.66 | 25.40 | 25.48 | 25.03 | 25.15 | 25.12 | 25.21 | 25.73 | 26.08 | 25.89 |
| **‘Fq1’-EF-1α** | 31.48 | 31.13 | 31.07 | 31.45 | 31.33 | 31.23 | 31.49 | 31.40 | 31.30 | 31.67 | 31.43 | 31.16 |
|  | 32.04 | 32.06 | 32.14 | 32.35 | 32.00 | 31.94 | 32.01 | 31.31 | 31.34 | 31.60 | 31.58 | 31.89 |
|  | 31.77 | 31.87 | 31.57 | 31.06 | 31.30 | 31.77 | 31.81 | 31.98 | 32.08 | 31.88 | 31.97 | 31.20 |
|  | 32.46 | 32.24 | 32.29 | 31.87 | 32.15 | 32.12 | 32.05 | 31.98 | 31.83 | 32.43 | 32.23 | 32.41 |

Supplemental Table S4. RT‒qPCR analysis of *OjPDS* expression levels in CMV-*OjPDS^N^*- or CMV-*OjPDS^C^*-infected ‘Yzcbq’.

| Plants | **CMV-*OjPDS^N^*** | | | | | | **CMV-*OjPDS^C^*** | | | | | |
| --- | --- | --- | --- | --- | --- | --- | --- | --- | --- | --- | --- | --- |
| **‘Yzcbq’-*OjPDS*** | 27.21 | 27.18 | 27.31 | 27.49 | 27.31 | 27.95 | 27.62 | 27.18 | 27.38 | 27.53 | 27.38 | 27.62 |
|  | 27.86 | 28.27 | 28.28 | 27.71 | 27.13 | 27.54 | 27.40 | 27.32 | 27.39 | 27.71 | 28.22 | 27.69 |
|  | 25.49 | 25.17 | 25.38 | 25.07 | 25.06 | 25.00 | 25.17 | 25.05 | 25.25 | 25.43 | 25.69 | 25.55 |
|  | 25.23 | 25.51 | 25.10 | 25.11 | 25.12 | 25.57 | 25.39 | 25.63 | 25.65 | 25.22 | 25.04 | 25.22 |
| **‘Yzcbq’-EF-1α** | 33.06 | 33.16 | 33.63 | 31.71 | 32.03 | 31.95 | 31.46 | 34.27 | 34.35 | 32.43 | 32.39 | 32.61 |
|  | 31.67 | 31.62 | 31.66 | 34.64 | 34.42 | 34.55 | 34.23 | 33.66 | 33.96 | 28.52 | 28.45 | 28.40 |
|  | 32.27 | 32.40 | 32.67 | 32.03 | 31.72 | 31.82 | 34.16 | 34.42 | 34.02 | 31.84 | 31.75 | 32.16 |
|  | 32.46 | 32.10 | 31.96 | 30.22 | 30.04 | 29.26 | 32.06 | 31.87 | 31.50 | 32.15 | 32.45 | 32.77 |

Supplemental Table S5. The silencing efficiency of *GID1* genes.

| Plants | No. of *GID1*  silenced/inoculated plants | Efficiency of  silencing *GID1* (%) |  |
| --- | --- | --- | --- |
| Fq1 |  |  |  |
| Repetition 1 | 5/8 | 62.5% |  |
| Repetition 2 | 3/8 | 37.5% |  |
| Repetition 3 | 4/8 | 50% |  |
| Yzcbq |  | | |
| Repetition 1 | 3/8 | 37.5% |  |
| Repetition 2 | 4/8 | 50% |  |
| Repetition 3 | 4/8 | 50% |  |
| *N. benthamiana* |  |  |  |
| Repetition 1 | 4/8 | 50% |  |
| Repetition 2 | 6/8 | 75% |  |
| Repetition 3 | 4/8 | 50% |  |

Supplemental Table S6. The silencing efficiency of miRNA319 genes.

| Plants | No. of miRNA319  silenced/inoculated plants | Efficiency of  silencing miRNA319 (%) |  |
| --- | --- | --- | --- |
| Fq1 |  |  |  |
| Repetition 1 | 6/8 | 75% |  |
| Repetition 2 | 4/8 | 50% |  |
| Repetition 3 | 3/8 | 37.5% |  |
| Yzcbq |  | | |
| Repetition 1 | 6/8 | 75% |  |
| Repetition 2 | 4/8 | 50% |  |
| Repetition 3 | 5/8 | 62.5% |  |
| *N. benthamiana* |  |  |  |
| Repetition 1 | 4/8 | 50% |  |
| Repetition 2 | 5/8 | 62.5% |  |
| Repetition 3 | 5/8 | 62.5% |  |

Supplemental Table S7. The silencing efficiency of miRNA396 genes.

| Plants | No. of miRNA396  silenced/inoculated plants | Efficiency of  silencing miRNA396 (%) |  |
| --- | --- | --- | --- |
| Fq1 |  |  |  |
| Repetition 1 | 5/8 | 62.5% |  |
| Repetition 2 | 5/8 | 62.5% |  |
| Repetition 3 | 4/8 | 50% |  |
| Yzcbq |  | | |
| Repetition 1 | 5/8 | 62.5% |  |
| Repetition 2 | 4/8 | 50% |  |
| Repetition 3 | 3/8 | 37.5% |  |
| *N. benthamiana* |  |  |  |
| Repetition 1 | 5/8 | 62.5% |  |
| Repetition 2 | 4/8 | 50% |  |
| Repetition 3 | 6/8 | 75% |  |

Supplemental Table S8. Primers used in this study.

| Primers | Primer secquences（5’→3’） |
| --- | --- |
| CMV-F | ATGGACAAATCTGAATCAACCAGT |
| CMV-R | ACTTTCTCATGTCACCTATATCAG |
| TuMV-F | CAAGCAATCTTTGAGGATTATG |
| TuMV-R | TATTTCCGATAAGCGAGAATA |
| Fny2b del BamSmaSpe-F | TCGGATCCCGGGACTAGTAACCTCCCCTTCCGCATCTC |
| Fny2b del BamSmaSpe-R | ATGGATCCTCAGACTCGGGTAACTCCG |
| pCB301CMV-Fny2-2b-F | GACAAACGTCGAACTCCAACTG |
| pCB301CMV-Fny2-2b-R | CGCGAGGAGGTGGAGATGCC |
| NbPDS-F | CGGGATCCAGCCCGTTGCTCAGTGTGTAC |
| NbPDS-R | GGACTAGTTTCTGTTTCGTGTAGTCACCAG |
| NbGID1-F | CGGGATCCCCAACTTCAAGTTGGCTTAC |
| NbGID1-R | GGACTAGTCCATCCATCATCATAAGCAC |
| qNbGA3oX1-F | GGGTTTCTTTCACATCCGCA |
| qNbGA3oX1-R | AAGTCAGGTCCAGACACCCT |
| qNbGA20oX1-F | AGCGTCCCTACGGAATTCAT |
| qNbGA20oX1-R | CGCCATTGGGTCCAATCTTC |
| qNbGAI-F | ATTCCAGGTGGAGCTGTGTTT |
| qNbGAI-R | TGTGCACAAGTCGAACACCA |
| NbEF1α-F | GCTGAACGTGAGCGTGGTAT |
| NbEF1α-R | GGGGGTGCATCTCAACAGATTTAAC |
| qNbTCP2-F  qNbTCP2-R  qNbTCP4-F  qNbTCP4-R  qNbPAO1-F  qNbPAO1-R  qNbU6-F  qNbU6-R | AAGTCGGATCAAGGCAAGGG  ATACCGCCTGCCAATAGCTC  CCGACAAGCCATCCTCTGC  CCTTTCCTTTGCCATTTCC  CGCTGCTCTGTCGTCATAGTC  CTTCCTTATCCTGCCACCTATC  GGAACGATACAGAGAAGATTAGCA  GTGCAGGGTCCGAGGT |
| qNbTCP2-R | ATACCGCCTGCCAATAGCTC |
| qNbTCP4-F | CCGACAAGCCATCCTCTGC |
| qNbTCP4-R | CCTTTCCTTTGCCATTTCC |
| qNbPAO1-F | CGCTGCTCTGTCGTCATAGTC |
| qNbPAO1-R | CTTCCTTATCCTGCCACCTATC |
| qNbU6-F | GGAACGATACAGAGAAGATTAGCA |
| qNbU6-R | GTGCAGGGTCCGAGGT |
| OjPDS^N^-F | CGGGATCCATTTCAATCTCCTACTCCCCCC |
| OjPDS^N^-R | GGACTAGTGTTTAATGGGTTAGGCGGACGG |
| OjPDS^C^-F | CGGGATCCAATGGATTTCACGCAGTGACTC |
| OjPDS^C^-R | GGACTAGTCCATTGAGGCTAGATACCTC |
| OjEF1α-F | GATGCCCCTGGACATCG |
| OjEF1α-R | CCTCATGTCCCTAACAGC |
| OjGID1-F | CGGGATCCCGCACATTCTTCAGCCAAC |
| OjGID1-R | GGACTAGTCCTTCAGGTAGATATGCTCTC |
| qOjGID1-F | CAGAGGGTGGTTCCTTTGAATACATG |
| qOjGID1-R | TTGACAGGAACCTTCCGATCAAGAAAC |
| qOjGA3oX1-F | TCGAGGCACTAGGCATAACC |
| qOjGA3oX1-R | TGACCCATCCGATGTCTTCC |
| qOjGA20oX1-F | CCACCTTGCCAGAAACCAGA |
| qOjGA20oX1-R | CTAGTATTAGCGGCTCCCCG |
| qOjGAI-F | GGCTCAATTCGCGGAAACAA |
| qOjbGAI-R | TCACCCTCGCGAAGATCAAG |
| qOjEF1α-F | AGCCTCTTCGTCTGCCACTTCA |
| qOjEF1α-R | GCTTCCAGGAGAGCCTCGTGAT  TCGAGGCACTAGGCATAACC  TGACCCATCCGATGTCTTCC  CCACCTTGCCAGAAACCAGA  CTAGTATTAGCGGCTCCCCG  GGCTCAATTCGCGGAAACAA  TCACCCTCGCGAAGATCAAG |
| miR319-F | CGGGATCCTTGGACTGAACT |
| miR319-R | GGACTAGTGGGAGCTCCCTA |
| mQ-miR319-F | CGCGTTGGACTGAAGGGA |
| Stem-loop-miR319-R | GTCGTATCCAGTGCAGGGTCCGAGGTATTCGCACTGGATACGACGGGAGC |
| qOjTCP2-F | GGCTTACGGAACTCCAATGG |
| qOjTCP2-R | AAGGTTGCTGGTGATGGTAATC |
| qOjTCP4-F | CAATGCTACTCCATCACCAAC |
| qOjTCP4-R | CCTCCTTCTACTCCACTACC |
| miR396-F | CGGGATCCTTCCACGGCTCT |
| miR396-R | GGACTAGTAGTTCAAGAATAGAGCCG |
| mQ-miR396-F | CGCGTTCCACGGCTTTCT |
| Stem-loop-miR396-R | GTCGTATCCAGTGCAGGGTCCGAGGTATTCGCACTGGATACGACAGTTCA |
| qOjGRF-9-like-F | CAGTGACTCCAGAACTTAGC |
| qOjGRF-9-like-R | CTCTCCTACACCGCCATC |
