# Supplementary material for: Cucumber mosaic virus-induced gene and microRNA silencing in water dropwort (Oenanthe javanica (Blume) DC)
Source: Plant Methods. 2024 Jan 11;20:6. doi: 10.1186/s13007-023-01129-4 (PMC10782793; doi:10.1186/s13007-023-01129-4)
Supplement: Supplementary file 2 — Additional file 2: Table S1. The silencing efficiency of PDS genes in N. benthamiana. Table S2. The silencing efficiency of PDS genes in water dropwort. Table S3. RT‒qPCR analysis of OjPDS expression levels in CMV-OjPDSN- or CMV-OjPDSC-infected ‘Fq1’. Table S4. RT‒qPCR analysis of OjPDS expression levels in CMV-OjPDSN- or CMV-OjPDSC-infected ‘Yzcbq’. Table S5. The silencing efficiency of GID1 genes. Table S6. The silencing efficiency of miRNA319 genes. Table S7. The silencing efficiency of miRNA396 genes. Table S8. Primers used in this study. [file 13007_2023_1129_MOESM2_ESM.docx]

The following materials are available in the online version of this article.

**
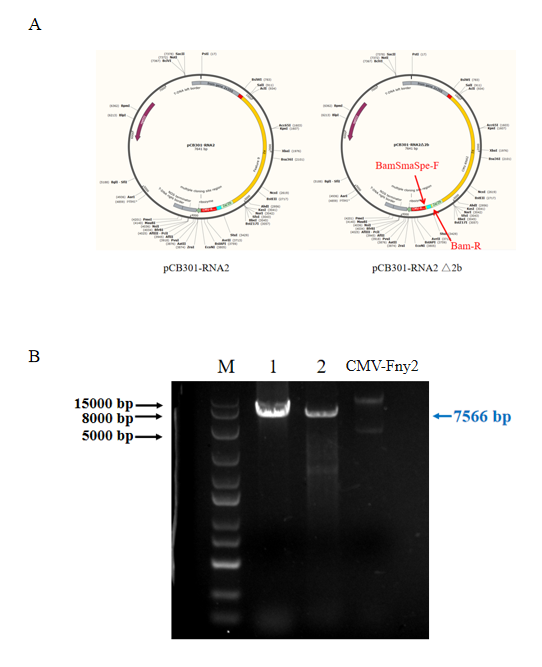
**

**Supplemental Figure S1.** Construction of pCB301-CMV-RNA2△2b. (A) Map of pCB301-RNA2 and pCB301-RNA2△2b. (B) Inverse-PCR detection of CMV-RNA2.

**
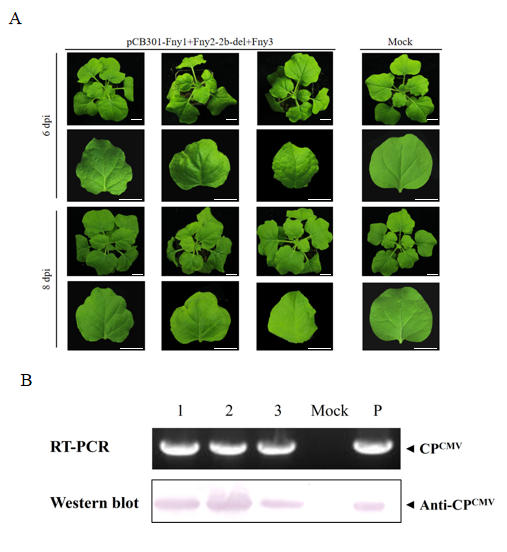
**

**Supplemental Figure S2.** The CMV-Fny 2b deletion mutant infected *N. benthamiana* by agro-infection. (A) Symptoms of the CMV-Fny 2b deletion mutant in *N. benthamiana*. Scale bars are 2 cm. (B) Detection of CMV-Fny 2b deletion mutant-infected *N. benthamiana* by agro-infection (8 dpi). The vector plasmid with the CMV RNA3 insert was amplified as the positive (lane P) control.

**
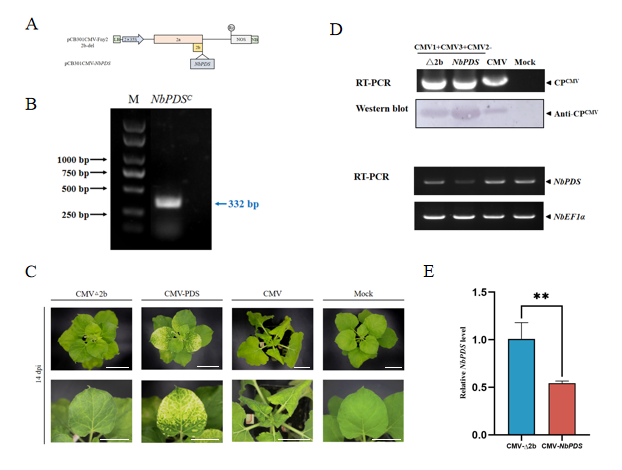
**

**Supplemental Figure S3.** Silencing of the *NbPDS* gene in *N. benthamiana* using the CMV VIGS vector. (A) Construct diagram of infectious clones of pCB301CMV-*NbPDS*. (B) RT‒PCR detection of the *PDS^C^* of *N. benthamiana*. (C) Phenotypes of pCB301CMV-*NbPDS* in *N. benthamiana* (14 dpi). Scale bars are 2 cm. (D) Detection of pCB301CMV-*NbPDS*-infected *N. benthamiana* by agro-infection (14 dpi). (E) RT‒qPCR analysis of *NbPDS* expression levels in CMV△2b- or CMV-*NbPDS*-infected plants.

**
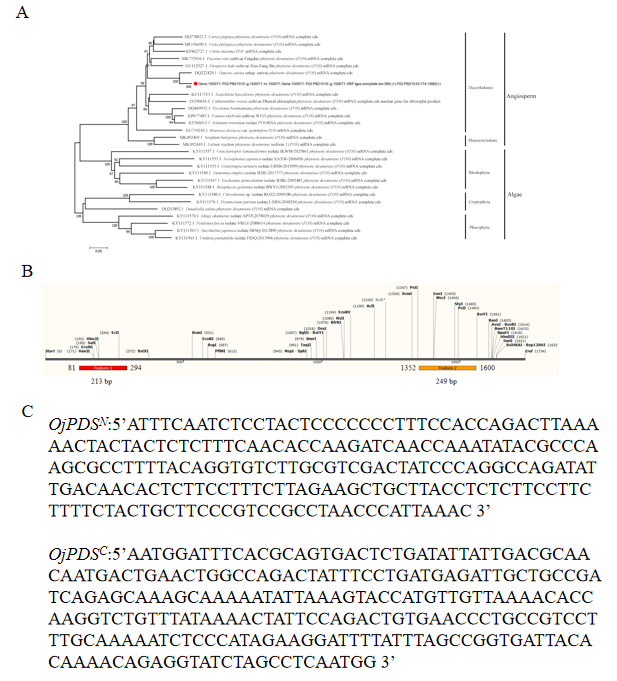
**

**Supplemental Figure S4.** Identification of *PDS* gene in *O. javanica*. (A) Phylogenetic analysis of *PDS* of *O. javanica*. (B) Map of *PDS* gene of *O. javanica*. (C) Silencing Sequence of *PDS* gene in *O. javanica*.

**
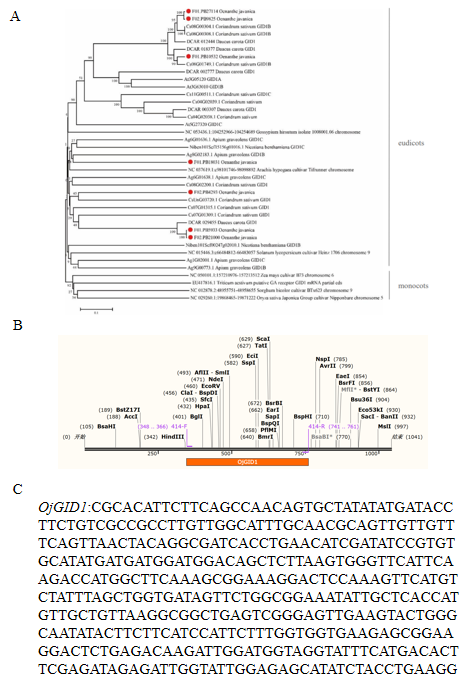
**

**Supplemental Figure S5.** Identification of *GID1* gene in *O. javanica*. (A) Phylogenetic analysis of *GID1* of *O. javanica*. (B) Map of *GID1* gene of *O. javanica*. (C) Silencing Sequence of *GID1* gene in *O. javanica*.

**
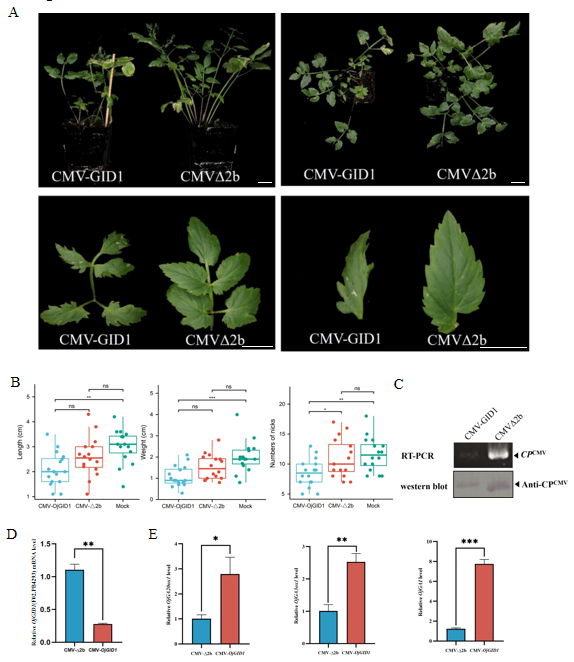
**

**Supplemental Figure S6.** Silencing of the *OjGID1* gene in *O. javanica* using the CMV VIGS vector. (A) Phenotypes of pCB301CMV-*OjGID1* in *O. javanica* (30 dpi). Scale bars are 2 cm. (B) Systematic leaf morphology analysis of pCB301CMV-*OjGID1* in ‘Fq1’ (30 dpi). (C) Detection of pCB301CMV-*OjGID1*-infected ‘Fq1’ by agro-infection (30 dpi). (D) RT‒qPCR analysis of *OjGID1* expression levels in CMV△2b- or CMV-*OjGID1*-infected plants. (E) RT‒qPCR analysis of GA-related genes expression in CMV△2b- or CMV-*OjGID1*-infected plants.

**
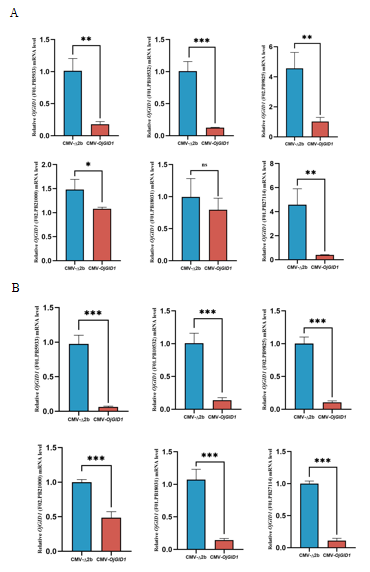
**

**Supplemental Figure S7.** RT‒qPCR analysis of 6 *OjGID1* genes expression in CMV△2b- or CMV-*OjGID1*-infected *O. javanica*. (A) RT‒qPCR analysis of 6 *OjGID1* genes expression in CMV△2b- or CMV-*OjGID1*-infected ‘Yzcbq’. (B) RT‒qPCR analysis of 6 *OjGID1* genes expression in CMV△2b- or CMV-*OjGID1*-infected ‘Fq1’.

**
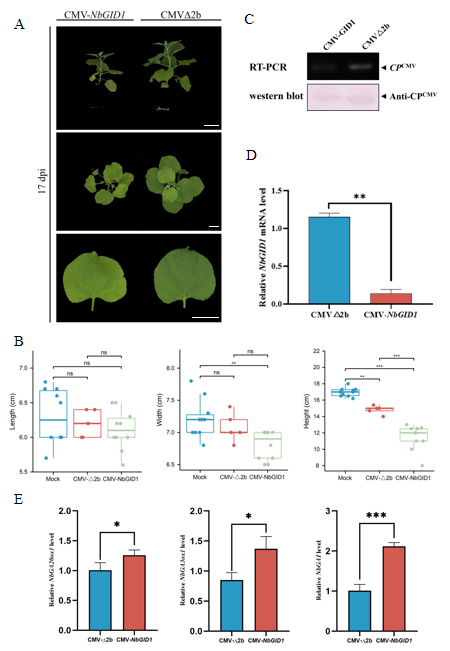
**

**Supplemental Figure S8.** Silencing of the *NbGID1* gene in *N. benthamiana* using the CMV VIGS vector. (A) Phenotypes of pCB301CMV-*NbGID1* in *N. benthamiana* (17 dpi). Scale bars are 2 cm. (B) Systematic leaf morphology analysis of pCB301CMV-*NbGID1* in *N. benthamiana* (17 dpi). (C) Detection of pCB301CMV-*NbGID1*-infected *N. benthamiana* by agro-infection. (D) RT‒qPCR analysis of *NbGID1* expression levels in CMV△2b- or CMV-*NbGID1*-infected plants. (E) RT‒qPCR analysis of *NbGID1* expression levels in CMV△2b- or CMV-*NbGID1*-infected plants.

**
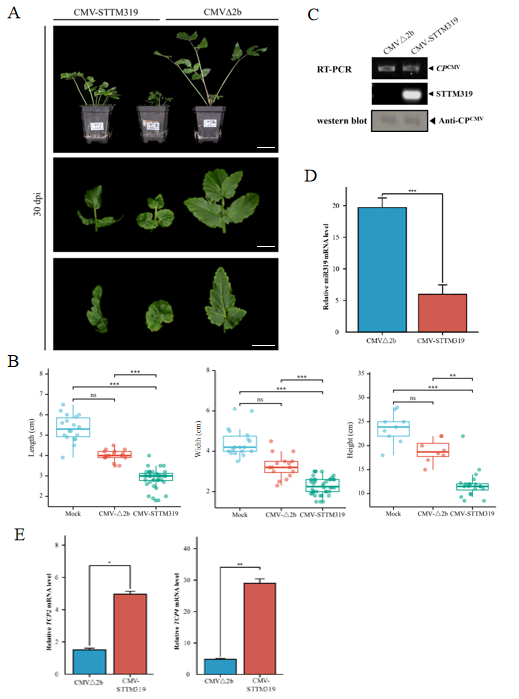
**

**Supplemental Figure S9.** Silencing of miR319 in *O. javanica* using CMV-based VbMS vector. (A) Phenotypes of plants inoculated with CMV-STTM319 at 30 dpi. Scale bars are 2 cm. (B) Systematic leaf morphology analysis of pCB301CMV-STTM319 in ‘Yzcbq’. (C) Detection of pCB301CMV-STTM319-infected ‘Yzcbq’ by agro-infection. (D) Stem‒loop RT‒qPCR analysis of miR319 expression levels in CMV△2b- or CMV-STTM319-infected plants. (E) Detection of the relative expression levels of the miR319 target genes *TCP2* and *TCP4*.

**
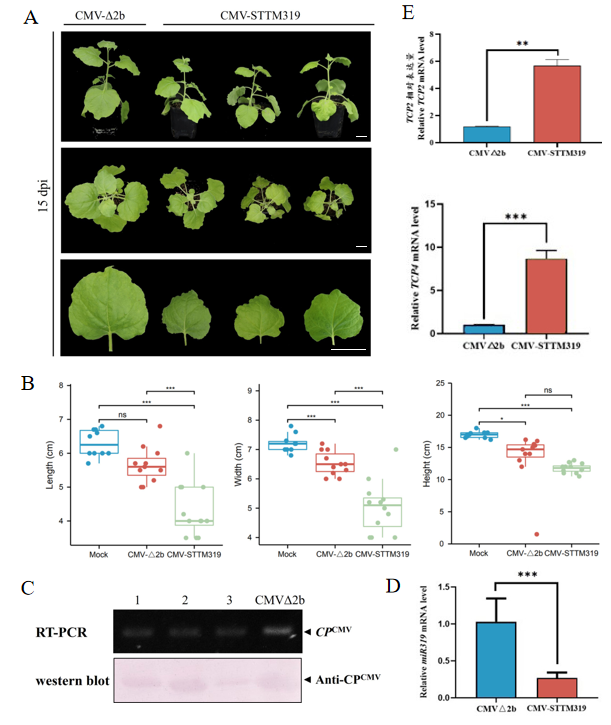
**

**Supplemental Figure S10.** Silencing of miR319 in *N. benthamiana* using CMV-based VbMS vector. (A) Phenotypes of plants inoculated with CMV-STTM319 at 15 dpi. Scale bars are 2 cm. (B) Systematic leaf morphology analysis of pCB301CMV-STTM319 in *N. benthamiana*. (C) Detection of pCB301CMV-STTM319-infected *N. benthamiana* by agro-infection. (D) Stem‒loop RT‒qPCR analysis of miR319 expression levels in CMV△2b- or CMV-STTM319-infected plants. (E) Detection of the relative expression levels of the miR319 target genes *TCP2* and *TCP4*.

**
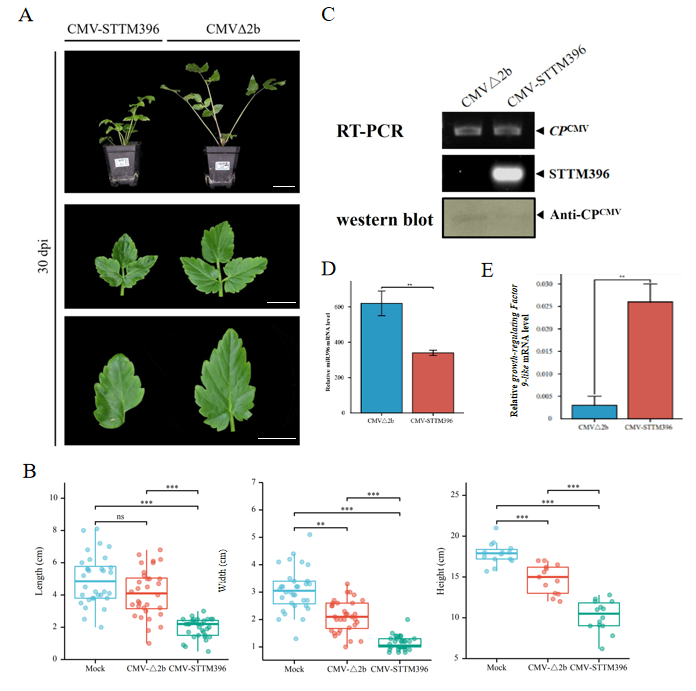
**

**Supplemental Figure S11.** Silencing of miR396 in *O. javanica* using CMV-based VbMS vector. (A) Phenotypes of plants inoculated with CMV-STTM396 at 30 dpi. Scale bars are 2 cm. (B) Systematic leaf morphology analysis of pCB301CMV-STTM396 in ‘Yzcbq’. (C) Detection of pCB301CMV-STTM396-infected ‘Yzcbq’ by agro-infection. (D) Stem‒loop RT‒qPCR analysis of miR396 expression levels in CMV△2b- or CMV-STTM396-infected plants. (E) Detection of the relative expression level of the miR396 target gene *growth-regulating Factor 9-like*.

**
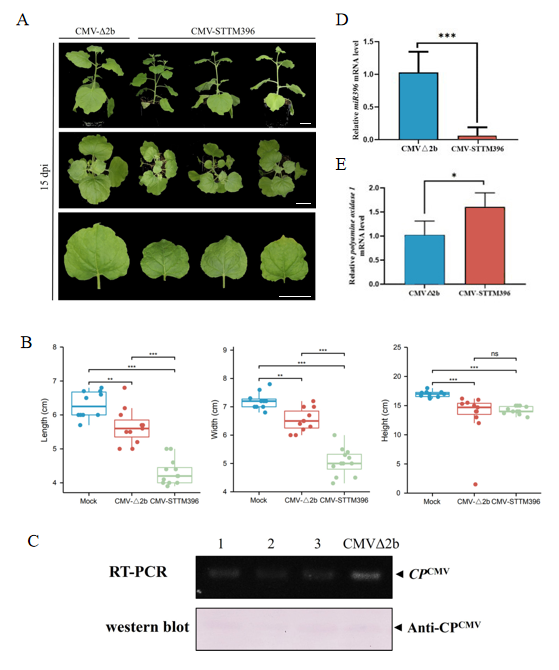
**

**Supplemental Figure S12.** Silencing of miR396 in *N. benthamiana* using CMV-based VbMS vector. (A) Phenotypes of plants inoculated with CMV-STTM396 at 15 dpi. Scale bars are 2 cm. (B) Systematic leaf morphology analysis of pCB301CMV-STTM396 in *N. benthamiana*. (C) Detection of pCB301CMV-STTM396-infected *N. benthamiana* by agro-infection. (D) Stem‒loop RT‒qPCR analysis of miR396 expression levels in CMV△2b- or CMV-STTM396-infected plants. (E) Detection of the relative expression level of the miR396 target gene *polyamine oxidase 1*.


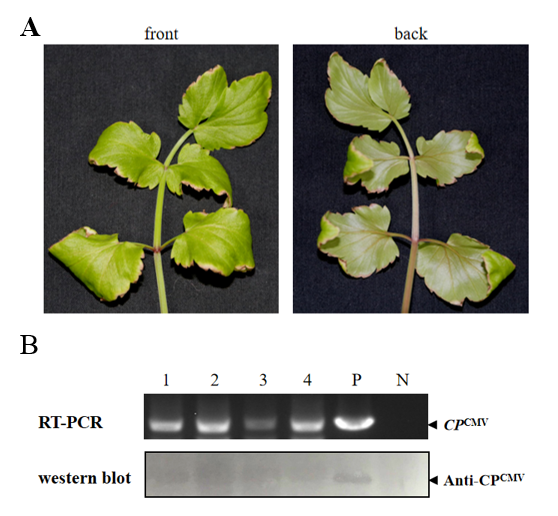


**Supplemental Figure S13.** Naturally infected of water dropwort by CMV. (A) Symptoms of CMV in O. javanica. (B) RT-PCR and western blot detection of the water dropwort samples.
